# Usefulness of ELISA Methods for Assessing LPS Interactions with Proteins and Peptides

**DOI:** 10.1371/journal.pone.0156530

**Published:** 2016-06-01

**Authors:** Victoria Martínez-Sernández, Ricardo A. Orbegozo-Medina, Fernanda Romarís, Esperanza Paniagua, Florencio M. Ubeira

**Affiliations:** Laboratorio de Parasitología, Departamento de Microbiología y Parasitología, Facultad de Farmacia, Universidad de Santiago de Compostela, Santiago de Compostela, Spain; nanyang technological university, SINGAPORE

## Abstract

Lipopolysaccharide (LPS), the major constituent of the outer membrane of Gram-negative bacteria, can trigger severe inflammatory responses during bacterial infections, possibly leading to septic shock. One approach to combatting endotoxic shock is to neutralize the most conserved part and major mediator of LPS activity (lipid A) with LPS-binding proteins or peptides. Although several available assays evaluate the biological activity of these molecules on LPS (e.g. inhibition of LPS-induced TNF-α production in macrophages), the development of simple and cost-effective methods that would enable preliminary screening of large numbers of potential candidate molecules is of great interest. Moreover, it would be also desirable that such methods could provide information about the possible biological relevance of the interactions between proteins and LPS, which may enhance or neutralize LPS-induced inflammatory responses. In this study, we designed and evaluated different types of ELISA that could be used to study possible interactions between LPS and any protein or peptide. We also analysed the usefulness and limitations of the different ELISAs. Specifically, we tested the capacity of several proteins and peptides to bind FITC-labeled LPSs from *Escherichia coli* serotypes O111:B4 and O55:B5 in an indirect ELISA and in two competitive ELISAs including casein hydrolysate (hCAS) and biotinylated polymyxin B (captured by deglycosylated avidin; PMX) as LPS-binding agents in the solid phase. We also examined the influence of pH, detergents and different blocking agents on LPS binding. Our results showed that the competitive hCAS-ELISA performed under mildly acidic conditions can be used as a general method for studying LPS interactions, while the more restrictive PMX-ELISA may help to identify proteins/peptides that are likely to have neutralizing properties *in vitro* or *in vivo*.

## Introduction

Lipopolysaccharide (LPS), which is the major constituent of the outer membrane of Gram-negative bacteria, typically consists of three regions: i) the hydrophobic domain lipid A, ii) a nonrepeating “core” oligosaccharide, and iii) a distal hydrophilic polysaccharide chain (O-antigen) composed by repeating oligosaccharide units [1,2]. The O-antigen may be absent in some deficient bacterial strains and is the main factor determining strain specificity. By contrast, lipid A is always present in Gram-negative bacteria and is responsible for the toxicity of LPS. When LPS is released from the bacterial surface into the bloodstream, it causes inflammation via activation of monocytes and endothelial cells, which can lead to septic shock and even death [3]. The discovery of new molecules that can bind and neutralize the toxicity of LPS is therefore of major interest in human therapy [4]. It may also be useful for developing new affinity sorbents for removal of endotoxin from the blood of patients with endotoxaemia [5] and for decontaminating biological products in the pharmaceutical industry [1].

LPS is a complex amphipathic molecule with a negative net charge and a high tendency to form micelles and even vesicles in water-based solutions [1]. It has been hypothesized that proteins and peptides with an exposed positively charged domain could interact with the negatively charged phosphoryl groups of LPS [6], thus promoting effective binding via electrostatic forces. Hydrophobic interactions involving the fatty acid residues of lipid A and hydrophobic amino acids have also been postulated to participate in the mechanism of LPS binding [7]. Naturally occurring proteins such as the bactericidal/permeability-increasing protein (BPI, human, ∽55 kDa, predicted pI = 9.4) and lactoferrin (LF, human, 82.4 kDa, pI = 8.7), which are produced by neutrophils, and the polymyxin B antibiotic (PMX, 1.2 kDa, pI = 8.9), produced by the bacterium *Paenibacillus* (*Bacillus*) *polymyxa*, are some examples of cationic molecules with proven LPS-binding ability that can reduce LPS activity. However, it has also been reported that nearly neutral proteins such as hemoglobin (HB, human, ∽64.5 kDa, pI = 6.8), the acute phase reactant LPS-binding protein (LBP, human ∽60 kDa, pI = 6.2) and the anionic human serum albumin (66.4 kDa, pI = 4.7) also bind LPS, but do not neutralize or even promote LPS toxicity [8–12]. This probably occurs via a mechanism that promotes disaggregation of supramolecular LPS structures [1]. The accumulated experience with different proteins and antimicrobial peptides seems to indicate that molecules with a net positive charge and an amphipathic character are more prone to binding LPS [4,13,14]. However, prediction of LPS binding for a particular peptide or protein is not a simple task as i) ionic and non-ionic driven forces (e.g. hydrophobic) are frequently implicated [15,16] and ii) the interactions may differ, considering the great diversity of O-antigens present in LPS structures [17].

The most widely used methods for testing LPS-binding activity of proteins and peptides include the classic *in vitro* assay used to detect tumor necrosis factor-alpha (TNFα) induced by LPS in mononuclear cells [18] and the *Limulus* amebocyte lysate assay [19]. Physicochemical methods, such as surface plasmon resonance [20], isothermal titration calorimetry [21], NMR spectroscopy [22] and methods using liquid crystals [23] have also been developed. Nonetheless, the use of ELISA techniques to test LPS-binding proteins and peptides is scarce, although these methods are less expensive and easily adaptable to the requirements of individual laboratories. Here we investigated the interactions between LPS and several proteins and peptides under different ELISA conditions, in order to overcome technical problems that may arise when analyzing such complex molecules. We found that some ELISAs can be used to investigate the LPS-binding ability of peptides or proteins while providing valuable information about the mode of interaction with LPS.

## Material and Methods

### Materials

FITC-labeled LPS from *Escherichia coli* serotypes O111:B4 and O55:B5, PMX sulphate salt, ovalbumin (OVA, grade V), bovine serum albumin (BSA, heat shock fraction), myoglobin from equine skeletal muscle (MYO), HB from bovine blood, LF from human milk, melittin from honey bee venom (MEL), histone f1 fraction from calf thymus (HF1) and SigmaFast OPD were purchased from Sigma-Aldrich (Madrid, Spain). Bovine casein (CAS, Hammarstein grade) was supplied by BDH Prolabo (VWR International Eurolab, Barcelona, Spain). Biotinylated PMX was obtained from Hycult Biotech (Uden, The Netherlands). Lysozyme from chicken egg white (LSZ) and deglycosylated avidin from hen egg white were procured from Fluka Analytical (Sigma-Aldrich, Steinheim, Germany). HRP-conjugated sheep anti-FITC was provided by Abd Serotec (Oxford, UK). Tween^®^ 20 (TW2) and Triton^®^ X-100 (TTX) were purchased from Merck KGaA (Darmstadt, Germany). Polystyrene microtiter plates were provided by Greiner Bio-One (Soria-Melguizo, Madrid, Spain). The synthetic peptide P3L (MCQCVQKYGTEFCKKRLA, 90% pure) from *Anisakis simplex* was synthesized at the Centro Nacional de Microbiología (Instituto de Salud Carlos III, Madrid, Spain). The synthetic protein corresponding to protein sMF6p/FhHDM-1 secreted by *Fasciola hepatica* (MF6p, gb CCA61804.1, 95% pure) was obtained from GeneCust Europe (Dudelange, Luxembourg).

### Indirect ELISAs

To determine the optimal conditions for this assays, we first investigated the influence on LPS binding of parameters such as the use of blocking agents and different buffers to dilute LPS. The wells of a polystyrene microtiter plate were coated with 100 μl of PMX and LSZ at 10 μg/ml in PBS and incubated for 2 h at 37°C. The plate was washed three times with PBS and blocked with 200 μl/well of 0.025% (50 μg/well) BSA or OVA in PBS for 1 h at RT. Empty wells were also blocked as controls. The plate was washed again and incubated with 100 μl/well of FITC-LPS (*E*.*coli* serotype O111:B4) at 1.25 μg/ml in PBS (0.15 M, pH 7.2) with 1 mM EDTA (PBS-EDTA) and PBS-EDTA containing 0.05% TW2 (PBS-EDTA/TW2) or 0.05% TTX (PBS-EDTA/TTX) for 30 min at RT, with shaking at 750 rpm on a microtiter plate shaker (orbit diameter: 1.5 mm). LPS was incubated in the presence of 1 mM EDTA in all assays to decrease its aggregation state and to prevent a “bridging effect” (promoted by divalent cations) between proteins and LPS [24,25]. The plate was washed five times with PBS-TW2 before addition of HRP-conjugated sheep anti-FITC diluted 1/4,000 in PBS-TW2 and incubation for 30 min at RT with shaking at 750 rpm. The plate was washed again with PBS-TW2 and incubated for 20 min at RT with 100 μl/well of substrate (SigmaFast OPD) before the reaction was stopped with 25 μl of 3 N H_2_SO_4_. The optical density (OD) was measured at 492 nm.

To further analyze the influence of detergents in the LPS dilution buffer, ELISA plates were coated with 200 μl/well of a casein hydrolysate (hCAS) solution (1.25% in PBS), prepared as previously described [26], for 2 hours at 37°C. The plates were washed three times with PBS and incubated after addition of 100 μl/well of FITC-LPS (*E*.*coli* serotype O111:B4) at a concentration of 5 μg/ml in PBS-EDTA or PBS-EDTA containing twofold dilutions of TW2 or TTX, starting at 0.05 and 0.1%(w/v), respectively. The plates were incubated for 30 min at RT with shaking at 750 rpm and then washed five times with PBS-TW2 before addition of 100 μl of HRP-conjugated sheep anti-FITC (1/4,000 in PBS-TW2) to each well and further incubation for 30 min at RT, with shaking at 750 rpm. Finally, the plates were washed again, incubated with OPD and the reaction was stopped as above.

**Fig 1:**
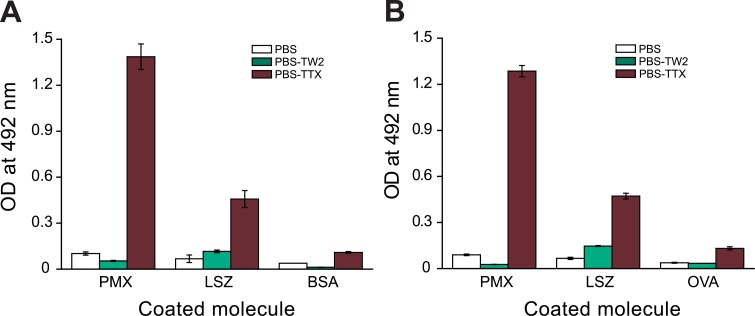
Comparison of LPS binding in indirect ELISA with different buffers and blocking agents. FITC-LPS from *E*. *coli* serotype O111:B4 was incubated at 1.25 μg/ml in PBS-EDTA (PBS), with or without 0.05% Tween 20 (PBS-TW2) or Triton X-100 (PBS-TTX), in wells coated with polymyxin B (PMX) or lysozyme (LSZ) (1 μg/well), or empty wells (control), which were previously blocked with BSA (A) or OVA (B). Each assay was performed in triplicate. Mean values ± SD are shown in the graph.

Once the optimal conditions for testing the proteins/peptides by indirect ELISA were determined (BSA as blocking agent and PBS-EDTA containing 0.05% TTX as incubation buffer for LPS), the other proteins/peptides were tested under the same conditions. Although BSA and OVA yielded similar results (Fig 1), BSA was chosen because of its extended use as blocking agent. The proteins/peptides were coated and blocked with BSA, and FITC-LPSs from *E*. *coli* serotypes O111:B4 (1.25 μg/ml) and O55:B5 (2.5 μg/ml) were incubated in PBS-EDTA/TTX following the same procedure as indicated above. The average OD value of the wells containing only BSA was subtracted from all OD values.

### Competitive hCAS-ELISA

A competitive indirect ELISA was performed to study the ability of different proteins/peptides to compete with hCAS for binding to LPS. Prior to carrying out the competitive ELISA, we tested the binding of LPS to hCAS-coated plates at two different pHs and at medium to high ionic strength. For this purpose, FITC-LPS (*E*. *coli* serotypes O111:B4 and O55:B5) at 5 μg/ml was diluted in PBS-EDTA, acetate buffer-EDTA (0.15 M, pH 5.6), or the same buffers containing 1M NaCl, and then incubated in hCAS-coated plates following the same steps as above.

The competitive ELISA was performed by preincubating FITC-LPSs from *E*. *coli* serotypes O111:B4 and O55:B5 (5 μg/ml) with four different concentrations of several proteins/peptides (40, 10, 1 and 0.25 μg/ml) or alone (controls) in PBS-EDTA, or acetate buffer-EDTA, for 1 hour at RT. The samples were then added (100 μl/well) to wells containing the hCAS and the plates were incubated for 30 min at RT with shaking at 750 rpm. HRP-conjugated sheep anti-FITC and OPD were added to wells after a washing step with PBS-TW2. The percentage inhibition of LPS binding to hCAS was calculated as follows: [(OD_control_-OD_test_/OD_control_)] x 100, where OD_test_ is the average OD_492_ of the wells containing LPS and the inhibitor, and OD_control_ is the average value for LPS alone. The average OD value of the wells without LPS (negative control) was subtracted from all OD values.

### Competitive PMX-ELISA

A competitive capture ELISA was performed to study the ability of different proteins/peptides to compete with PMX for binding to LPS. Plates were coated with 100 μl of 10 μg/ml deglycosylated egg white avidin in PBS for 2 hours at 37°C, washed three times with PBS and blocked with 1% BSA in PBS (200 μl/well) for 1 hour at RT. The plates were washed three times with PBS and incubated with biotinylated PMX diluted 1/50 in PBS containing 0.1% BSA (PBS-BSA) for 1 hour at RT under shaking at 750 rpm. FITC-LPSs from *E*. *coli serotypes* O111:B4 (1.25 μg/ml) and O55:B5 (2.5 μg/ml) were preincubated with three concentrations of the proteins/peptides (10, 1 and 0.25 μg/ml) or alone (control) in PBS-EDTA/BSA for 1 hour at RT. The plates were then washed five times with PBS, samples were added (100 μl/well) and the plates were incubated for 30 min at RT with shaking at 750 rpm. The plates were washed five times with PBS-TW2, 100 μl/well of HRP-conjugated sheep anti-FITC (1/4,000 in PBS-BSA) was added and the plates were incubated for 30 min at RT with shaking at 750 rpm. Plates were then washed, OPD substrate was added and the reaction was stopped as previously indicated. The percentage inhibition of LPS binding to captured PMX was calculated as follows: [(OD_control_-OD_test_/OD_control_)] x 100, where OD_test_ is the average OD_492_ of the wells containing LPS and the inhibitor, and OD_control_ is the average OD_492_ of LPS alone. The mean OD values of wells with all reagents except for biotinylated PMX (negative control) was subtracted from all OD values.

### Statistical analysis

A student’s *t* test and variance coefficients were used to determine the significance of differences between inhibition and control values (without inhibition) in competitive ELISAs. All statistical analyses were conducted using the GradPad Instat statistical package (GraphPad Software Inc, CA, USA). Differences were considered significant at *p* <0.05.

## Results

### Testing LPS-binding activity of proteins and peptides in indirect ELISA

In indirect ELISAs, the target proteins or peptides of interest are typically immobilized on ELISA plates and the putative remaining reactive groups are blocked with an “irrelevant” protein to avoid non-specific binding (NSB) to the plate of the subsequently used immune reactants. However, when testing large amphoteric molecules such as LPSs, which form micelles and vesicles in aqueous solutions, direct interaction between LPS and the target protein may be hampered by the presence of the blocking agent due to steric hindrance. To evaluate the relevance of this phenomenon, we tested the ability of FITC-LPS to bind to PMX and LSZ in indirect ELISA with BSA (Fig 1A) or OVA (Fig 1B) as blocking agents (50 μg/well) and PBS-EDTA, PBS-EDTA/TW2 or PBS-EDTA/TTX buffers to dilute the FITC-LPS. As expected, the presence of a blocking agent impeded the binding of FITC-LPS incubated in PBS-EDTA to the LPS-binding molecules (PMX and LSZ), and the effect could not be prevented by addition of TW2 to the incubation buffer. However, steric hindrance by BSA or OVA was not observed when FITC-LPS was incubated in PBS buffer containing TTX.

**Fig 2:**
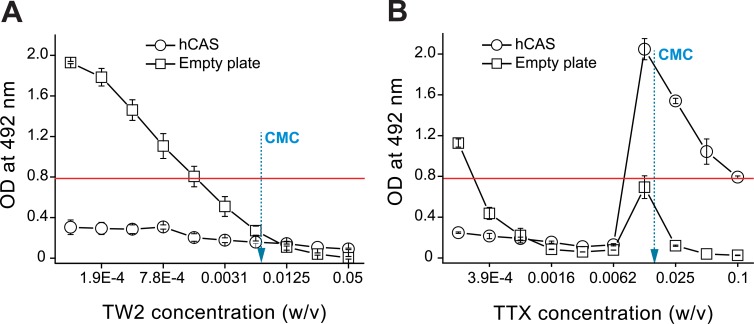
Influence of detergents on LPS binding to casein hydrolysate (hCAS)-blocked or empty plates. FITC-LPS isolated from *E*. *coli* serotype O111:B4 was incubated at 5 μg/ml in PBS-EDTA (control, red line) or in PBS-EDTA supplemented with twofold dilutions of Tween 20 (TW2, A) or Triton X-100 (TTX, B), starting at concentrations of 0.05 and 0.1% (w/v) respectively. Samples were tested in triplicate (mean values ± SD). CMC: critical micelle concentration (0.0074 and 0.0155% w/v, for TW2 and TTX, respectively).

For a more detailed evaluation of the different effects of TW2 and TTX detergents on the binding between LPS and LPS-binding proteins, we tested several concentrations of TW2 and TTX, below and above their CMC, on the binding of FITC-LPS to empty plates and to ELISA plates saturated with 1.25% hCAS. A casein hydrolysate solution was chosen as a protein target because of its excellent quality as a blocking agent in ELISA [27] and because it binds efficiently to FITC-LPS [28]. Independently of the concentration used, TW2 (CMC; 0.0074% w/v) was able to inhibit the binding of FITC-LPS to hCAS, while, as expected, concentrations of about 0.0125% (above the CMC) were required to completely inhibit the binding of FITC-LPS to empty wells (Fig 2A). On the contrary, TTX (CMC = 0.0155%) strongly promoted binding of FITC-LPS to hCAS in the range of 0.012 to 0.1% (close to and above the CMC), while at lower concentrations it showed an inhibitory effect (Fig 2B). In comparison with TW2, TTX showed a broader range of inhibition of binding of FITC-LPS to empty wells, except at a concentration of 0.012%, at which an increase in the LPS binding was observed.

**Fig 3:**
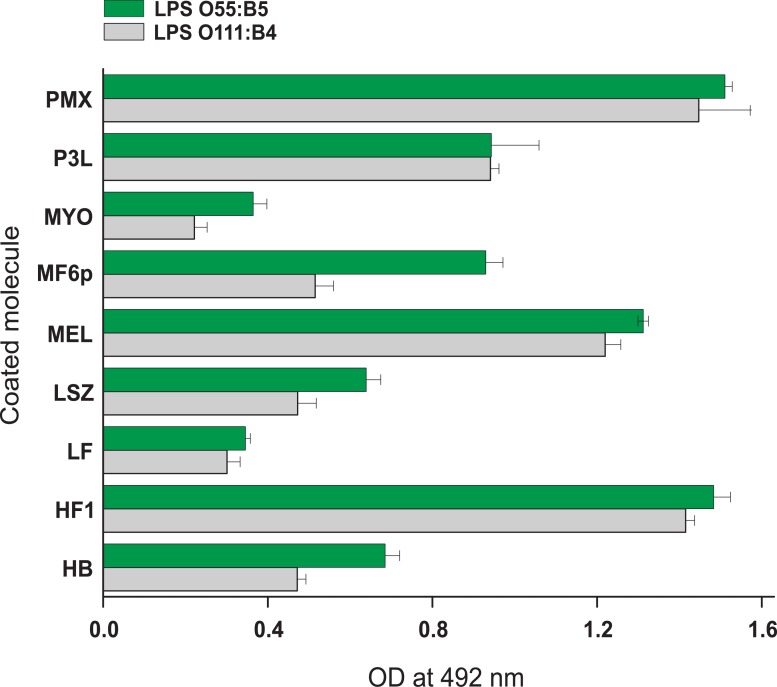
LPS binding in indirect ELISA. FITC-LPSs from *E*. *coli* serotypes O111:B4 (1.25 μg/ml) and O55:B5 (2.5 μg/ml), diluted in PBS-EDTA with 0.05% Triton X-100, were added to plates coated with several proteins and peptides (1 μg/well) and blocked with BSA. Data represent the mean values ± SD of duplicate samples. HB, hemoglobin; HF1, histone f1 fraction; LF, lactoferrin; LSZ, lysozyme; MEL, melittin; MF6p, synthetic FhHDM-1/MF6p; MYO, myoglobin; P3L, *A*. *simplex* peptide: MCQCVQKYGTEFCKKRLA; PMX, polymyxin B; BSA, bovine serum albumin.

Taking into account the promoter effect of TTX on the binding of FITC-LPS to LPS-binding proteins, we investigated the LPS-binding properties of several proteins and peptides in the presence of this detergent. We used TTX at a concentration of 0.05% to guarantee a good ELISA signal and adequate inhibition of NSB, as previously shown by testing FITC-LPS-binding on empty wells (see Fig 2B). Several cationic, nearly neutral and anionic proteins and peptides, and two FITC-LPS conjugates corresponding to *E*. *coli* serotypes O111:B4 and O55:B5 were used in the study. We observed that, with the exception of BSA (used as a blocking agent), all proteins and peptides tested bound more or less strongly to both FITC-LPS conjugates, although OD was maximal with PMX, MEL, HF1 and the *Anisakis* peptide P3L (Fig 3). Although P3L has not previously been classified as a LPS-binding molecule, it may have reacted in this assay as a result of its cationic nature.

### Testing LPS-binding activity of proteins and peptides in a competitive hCAS-ELISA

**Fig 4:**
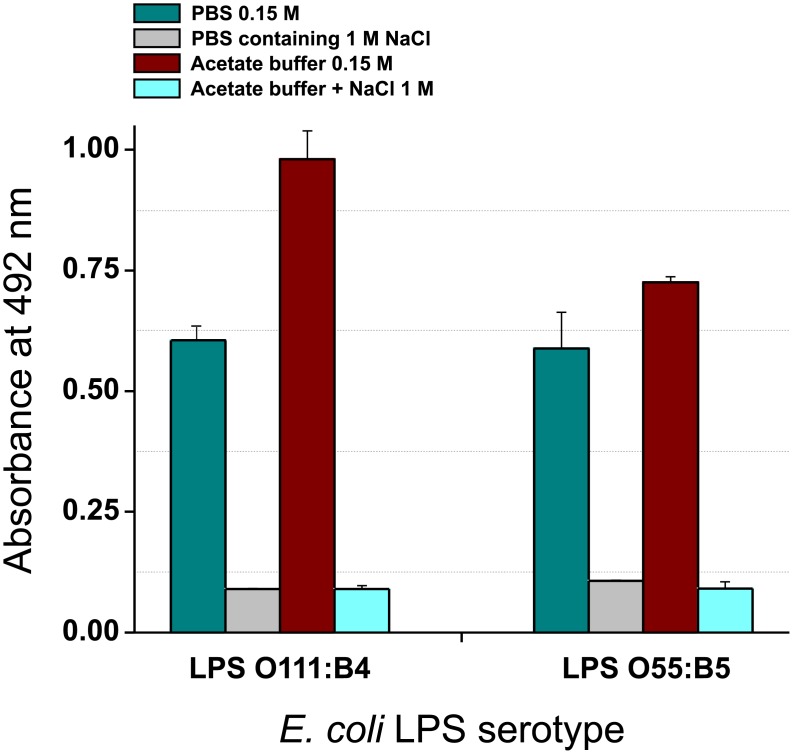
Influence of pH and ionic strength on LPS binding to casein hydrolysate (hCAS)-coated plates. FITC-LPSs from *E*. *coli* serotypes O111:B4 and O55:B5, diluted at 5 μg/ml in PBS-EDTA (pH 7.2) and acetate buffer-EDTA (pH 5.6) prepared 0.15 M, or the same buffers containing 1M NaCl, were added to hCAS-coated plates.

In previous studies [26,28] we have observed that a 1.25% dilution of hCAS forms a bed in the wells of ELISA plates to which the FITC-LPS binds. In the present study, we tested the influence on binding of the LPS serotype as well as the pH and ionic strength of the incubation buffer. The interaction between hCAS and LPS increased significantly under mildly acidic conditions (acetate buffer, pH 5.6) relative to neutral conditions (PBS, pH 7.2), and the interaction was not dependent on the LPS serotype (Fig 4). Moreover, electrostatic forces appear to be involved, as binding could be prevented by adding 1M NaCl to each of the incubation buffers tested. Taking this into account, we designed a competitive ELISA to investigate the ability of proteins and peptides to inhibit binding of FITC-LPS to hCAS. As interactions between proteins or peptides and LPS may produce different results depending on the concentrations used, we tested each in the broad range of 0.25 to 40 μg/ml. Higher concentrations were avoided as excess protein may cause non-specific inhibition due to steric hindrance, especially when testing large molecules. For comparison, we used two FITC-LPS conjugates (LPS *E*. *coli* serotypes O111:B4 and O55:B5) and both incubation buffers (PBS and acetate buffer). The expected outcomes for these experiments were: i) a decrease in OD values (i.e. an inhibitory profile), when the molecule tested binds to LPS and competes with hCAS, ii) an increase in OD values (i.e. enhancement profile), when the molecule tested promotes LPS binding to hCAS, and iii) no effect, indicating that the protein/peptide tested does not interfere with LPS binding to hCAS.

**Fig 5:**
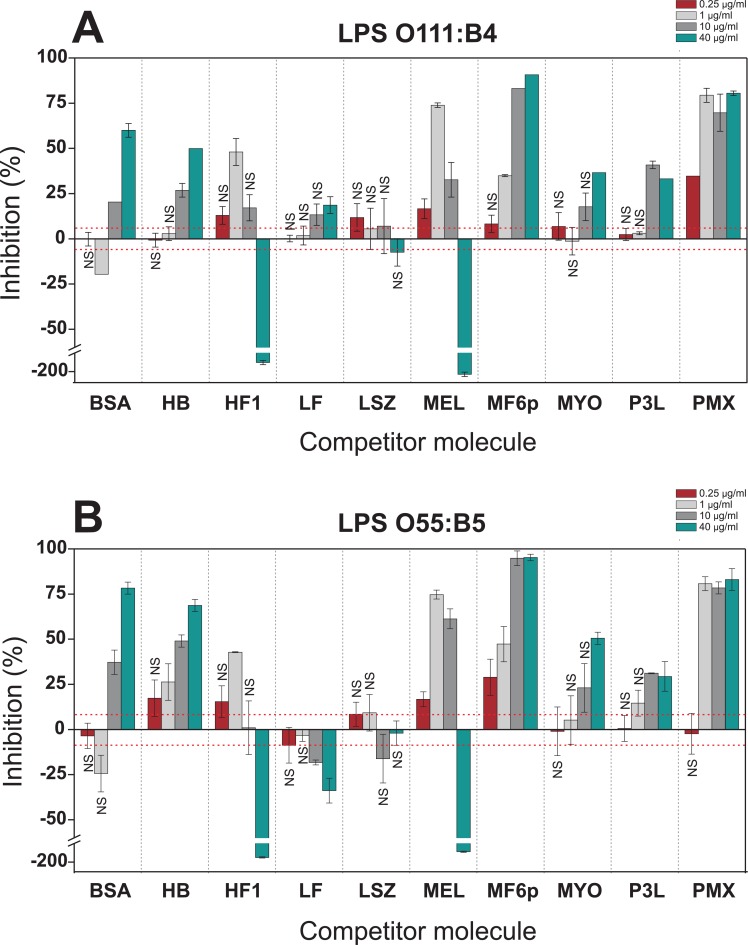
Competitive hCAS-ELISA performed under neutral conditions. FITC-LPSs (5 μg/ml) from *E*. *coli* serotypes O111:B4 (A) and O55:B5 (B) were preincubated with different concentrations of proteins and peptides (40, 10, 1 and 0.25 μg/ml) in PBS-EDTA (pH 7.2) and added to the wells coated with casein hydrolysate (hCAS). Data are expressed as percentage inhibition of LPS binding to hCAS by the target molecule and are the mean values ±SD of duplicate wells. The average values of the optical density (492 nm) of control wells (without inhibitor) were 0.311 (±0.019, red dashed line) and 0.300 (±0.025, red dashed line) for serotypes O111:B4 and O55:B5, respectively. Differences were considered significant at *p* <0.05. NS: not significant. BSA, bovine serum albumin; HB, hemoglobin; HF1, histone f1 fraction; LF, lactoferrin; LSZ, lysozyme; MEL, melittin; MF6p, synthetic FhHDM-1/MF6p; MYO, myoglobin; P3L, *A*. *simplex* peptide: MCQCVQKYGTEFCKKRLA; PMX, polymyxin B.

**Fig 6:**
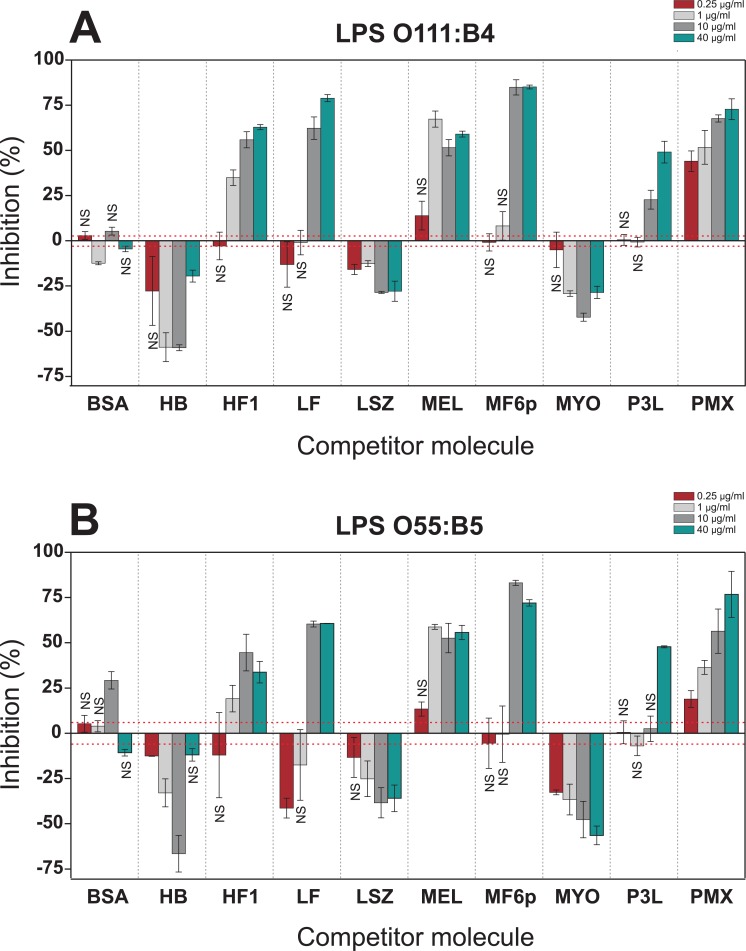
Competitive hCAS-ELISA performed under acidic conditions. FITC-LPSs (5 μg/ml) from *E*. *coli* serotypes O111:B4 (A) and O55:B5 (B) were preincubated with several concentrations of proteins and peptides (40, 10, 1 and 0.25 μg/ml) in acetate buffer-EDTA (pH 5.6) and added to casein hydrolysate (hCAS)-coated wells. Data are expressed as percentage inhibition of LPS binding to hCAS by the target molecule. Results are the means ±SD of duplicate wells. The average values of the optical density (492 nm) of control wells (without inhibitor) were 1.009 (±0.030, red dashed line) and 0.746 (±0.046, red dashed line) for serotypes O111:B4 and O55:B5, respectively. Differences were considered significant at *p* <0.05. NS: not significant. BSA, bovine serum albumin; HB, hemoglobin; HF1, histone f1 fraction; LF, lactoferrin; LSZ, lysozyme; MEL, melittin; MF6p, synthetic FhHDM-1/MF6p; MYO, myoglobin; P3L, *A*. *simplex* peptide: MCQCVQKYGTEFCKKRLA; PMX, polymyxin B.

When the FITC-LPS was incubated with different proteins/peptides at neutral pH (PBS; Fig 5A and 5B), certain proteins/peptides produced opposite results depending on their concentration and LPS-serotype. Note, for example, that for MEL or HF1 an inhibitory profile was observed in the range of 0.25–10 μg/ml, but at the highest concentration (40 μg/ml) both proteins clearly enhanced the ELISA signal. In addition, the LF produced moderate inhibition (Fig 5A) or enhancement (Fig 5B) of OD values depending on the LPS serotype. This variability was not observed under mildly acidic conditions (acetate buffer; Fig 6A and 6B), in which the higher OD values obtained for the control wells (no inhibitor; OD = 1.009 at pH 5.6 vs. 0.311 at pH 7.2 for serotype O111:B4, and OD = 0.746 vs. 0.300 for serotype O55:B5) produced much more reliable and consistent results. As shown in Fig 6, under mildly acidic conditions, six molecules (PMX, P3L, MF6p, MEL, LF and HF1) exerted a predominant inhibitory effect, while three molecules (MYO, HB and LSZ) enhanced the OD values.

### Testing LPS-binding activity of proteins and peptides in a competitive PMX-ELISA

**Fig 7:**
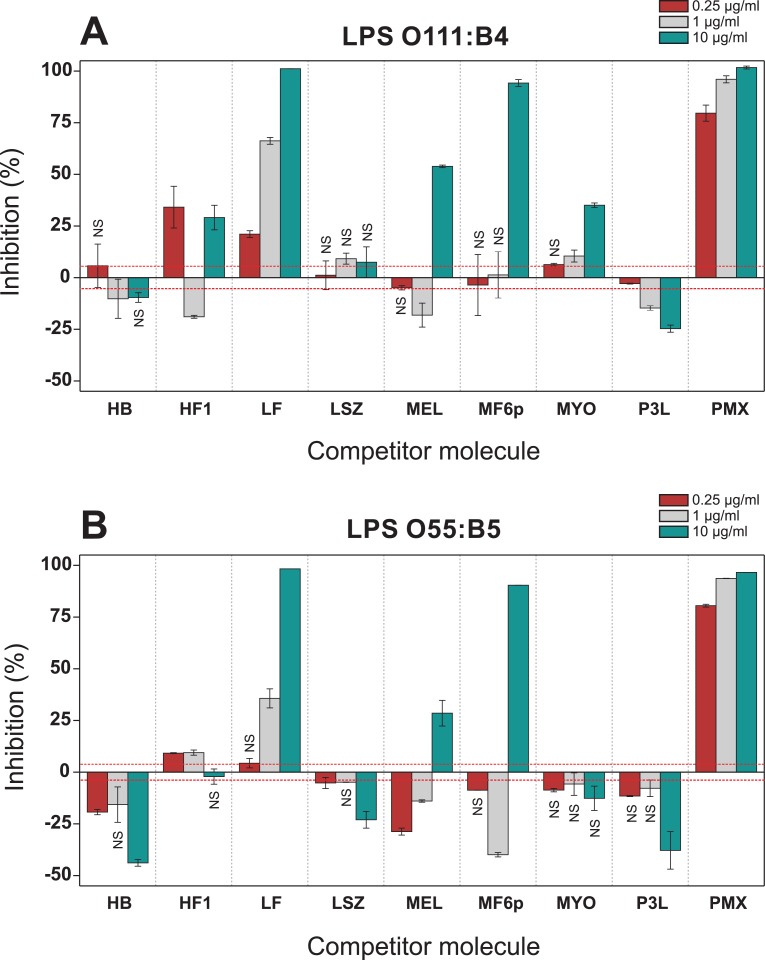
Competitive PMX-ELISA. FITC-LPSs from *E*. *coli* serotypes O111:B4 (A) and O55:B5 (B) were preincubated, at 1.25 and 2.5 μg/ml, respectively, with three different concentrations of proteins and peptides (10, 1 and 0.25 μg/ml) in PBS-EDTA with 0.1% BSA and added to wells containing biotinylated polymyxin B captured by deglycosylated avidin. Results are expressed as percentage inhibition of LPS binding to captured polymyxin B by the target protein/peptide, and are the mean values ±SD for duplicate wells. The average optical densities (492 nm) of control wells (without inhibitor) were 0.904 (±0.042, red dashed line) and 0.842 (±0.036, red dashed line) for serotypes O111:B4 and O55:B5, respectively. Differences were considered significant at *p* <0.05. NS: not significant. HB, hemoglobin; HF1, histone f1 fraction; LF, lactoferrin; LSZ, lysozyme; MEL, melittin; MF6p, synthetic FhHDM-1/MF6p; MYO, myoglobin; P3L, *A*. *simplex* peptide: MCQCVQKYGTEFCKKRLA; PMX, polymyxin B.

It has previously been proposed that direct interaction or blocking of the PMX binding site is required to neutralize the toxicity of LPS, thus preventing LPS-induced B cell and macrophage activation [29]. If this were the case, only proteins/peptides with this property would be of interest in the search for new LPS-binding molecules that could prevent LPS toxicity. Considering this, we designed a competitive capture ELISA to investigate the ability of different peptides and proteins (tested in the range of 0.25–10 μg/ml) to inhibit the binding of FITC-LPS (*E*. *coli* serotypes O111:B4 and O55:B5) to biotinylated PMX captured by deglycosylated avidin. In this ELISA, BSA was used to block the avidin-coated plates and in all incubation steps. As shown in Fig 7, three molecules (LF, MEL and MF6p) strongly inhibited the binding of both FITC-LPSs serotypes to captured PMX, although MF6p and MEL only caused inhibition at a concentration of 10 μg/ml. We also observed that MYO and HF1 partly inhibited the binding of FITC-LPS O111:B4 (Fig 7A), but not the O55:B5 serotype (Fig 7B), to PMX, although HF1 displayed an enhancing effect at 1 μg/ml. Finally, low to mild enhancement of OD values was observed with HB, LSZ and P3L, but with the exception of P3L, this was only evident with serotype O55:B5. The differences between serotypes are probably due to the addition of a higher concentration of serotype O55:B55 (2.5 μg/ml) than of serotype O111:B4 (1.25 μg/ml) in order to yield comparable OD values in control wells.

## Discussion

In this study we designed and evaluated the usefulness of several new ELISAs for assessing LPS binding to several proteins and peptides, most of which have previously been reported to interact with LPS. Almost all reported ELISAs for studying LPS-binding interactions of peptides or proteins use capture strategies [30] or are indirect ELISAs based on direct immobilization of LPS to the plates [31,32]. Unfortunately, a major disadvantage of these strategies is that a primary or a secondary antibody (either monoclonal or polyclonal) is required to recognize the target molecule directly or through a label. As this makes general use of these methods difficult, mainly when numerous molecules are assayed, we investigated ELISA methods that may be suitable for application to any peptide or protein.

Initially, we evaluated whether an indirect ELISA with the protein/peptide of interest immobilized on the plate could be used to assess LPS binding. We observed that the presence of a blocking agent (e.g. BSA and OVA) impeded binding of LPS diluted in PBS to the plates coated with the target protein/peptide (Fig 1). This can probably be explained by steric hindrance promoted by the blocking protein, due to the large size and aggregate structure of LPS in aqueous solutions. This is consistent with the fact that when the blocking step is omitted, FITC-LPS is able to bind to wells coated with an excess of the target molecules (not shown). Moreover, when the protein of interest is indirectly attached to the plastic surface by another molecule (e.g. a specific antibody or a spacer arm), such as in the competitive PMX-ELISA proposed, binding to LPS takes place regardless of the presence of a blocking agent.

One way of solving the steric problem is to disaggregate LPS molecules by using non-ionic detergents such as TW2 and TTX [15,31,33], which are also used in immunoassays to disrupt non-specific low-affinity interactions [34–36]. However, of the detergents tested, only TTX promoted binding of FITC-LPS to the target peptide/protein immobilized in the ELISA plate and, as expected, this effect only occurred at concentrations equal to or exceeding the CMC (Figs 1 and 2), which is necessary for a detergent to be capable of solubilizing amphipathic molecules [37]. Monomers of TTX were unable to destabilize LPS aggregates sufficiently, but also impeded binding of LPS to hCAS (Fig 2B).

Regarding the usefulness of this ELISA format for studying LPS binding, the results obtained with TTX (Fig 3) indicate that positively charged peptides and highly basic proteins (see Table 1) are more prone to binding to FITC-LPS than nearly neutral (e.g. MYO and HB) or acidic (e.g. BSA) molecules. The use of detergents (TTX to incubate LPS and TW2 in subsequent steps) may impede or break important interactions between LPS and proteins or peptides as LPSs are accommodated in the micelles by non-polar interactions of acyl chains of lipid A with the detergent, and they are consequently separated from the water phase [15]. In such a scenario, electrostatic forces between positive residues of the proteins/peptides and the negative groups of LPS would be favored over other types of interactions (e.g. hydrophobic interactions with acyl chains of lipid A). Moreover, as the target molecules are randomly coupled to the plates in indirect ELISA, some key residues may not be available for binding to LPS. This may lead to erroneous conclusions about the LPS-binding nature of some molecules. Consequently, we do not recommend this type of indirect ELISA for testing the ability of proteins and peptides to bind LPS. Moreover, these methods do not provide any additional information about the mode of interaction between a given peptide or protein and LPS.

**Table 1:** LPS-binding properties of proteins and peptides investigated in the study.

Protein/peptide	MW (kDa)	pI	Effect on LPS induced responses	References
Serum albumin (bovine)	66.5	4.7	Binding/enhancing^b^	[8,12]
Casein (bovine)	19–25	4.1–5.8	Binding/neutralizing (casein derived peptides)	[38,39]
Hemoglobin (bovine)	64.5	6.8	Binding/enhancing^b^	[9,11,25]
Histone f1 (bovine)	21.5	>10	Binding (H1, H2A, H2B, H3 and H4)/neutralizing (only tested with H2A)	[40]
Lactoferrin (human)	82.4	8.7	Binding/neutralizing	[41,42]
Lysozyme (egg white)	14.3	11.4	Binding/neutralizing (dependent on LPS serotype)	[29,43,44]
sMF6p/FhHDM-1	7.8	9.57^a^	Binding/neutralizing	[45]
Melittin	2.8	12^a^	Binding/neutralizing	[46]
Myoglobin (equine)	17.6	7.3 and 6.8	*nd*	-
Ovalbumin	44.3	4.5–4.9	No interaction	[12,47]
P3L	2.1^a^	9.1^a^	*nd*	-
Polymyxin B	1.2	8.9	Binding/neutralizing	[48,49]

^a^The theoretical molecular weight (MW) and the isoelectric point (pI) were computed using the ExPASy ProtParam tool

^b^Most studies were performed with the human derivative

*nd*: not determined

In contrast to indirect methods, competitive ELISAs lack most of the above mentioned disadvantages since i) the target peptides and proteins are tested in a free soluble state, and all residues are therefore available, ii) they can be tested at several concentrations relative to LPS, and iii) the inhibition procedure is not affected by washing steps. However, some difficulties may also arise in finding the appropriate LPS-binding molecule for immobilization on the ELISA plate and in choosing the optimal conditions for carrying out the assay. In this study, we investigated the usefulness of two competitive ELISAs that include hCAS and biotinylated PMX (captured by deglycosylated avidin) in the solid phase. Regarding hCAS, in addition to yielding excellent blocking of NSB in ELISA and the fact that some of the generated peptides are able to bind FITC-LPS, LPS-binding peptides derived from bovine casein have been reported to be capable of inhibiting the LPS-stimulated inflammatory response [38,39]. The lipopeptide PMX was selected as it is considered the “gold standard” for LPS-sequestering agents and its mechanism of interaction with LPS has been well studied [49]. However, unlike hCAS, which has both LPS-binding and NSB blocking properties, for PMX it was first necessary to capture the biotinylated molecule with avidin, and to use BSA to block the plates and in all subsequent incubation steps.

As already mentioned, when the competitive hCAS-ELISA was performed at pH 7.2 (PBS), some LPS-binding molecules produced opposite results depending on the protein concentration and the serotype of the LPS tested. In addition, some molecules, such as LF and LSZ, displayed lower LPS-binding activity than expected from previously reported data (Fig 5). We also observed significant inter-assay variation in LPS binding to hCAS (without inhibitor) that may have influenced the inhibition results. For example, considering serotype O111:B4, OD values of 0.6 and 0.3 were obtained for hCAS in the experiments summarized in Figs 4 and 5, respectively. However, when inhibition was carried out in mildly acidic buffer (i.e. acetate buffer; Fig 6), the molecules exhibited the same behavior for both LPSs and in a concentration dependent manner. Moreover, binding of FITC-LPS to hCAS in the absence of any competitor increased at acidic pH (Fig 4), resulting in less variable results and less dependency on external variables (e.g. incubation time, temperature, shaking, etc.). The higher signal obtained with hCAS under acidic pH can be attributed to an increase in electrostatic forces between positively charged CAS-derived peptides and the negatively charged LPS groups (pI = 1.3). The use of acidic conditions was previously used to investigate the relevance of electrostatic interactions between peptides and LPS [25] and to determine the best conditions for removing endotoxins from samples to maximize protein recovery [16]. We observed that some of the proteins/peptides tested interacted more strongly with LPS at pH 5.6 than at pH 7.2. Thus, lowering the pH may facilitate the detection of LPS-binding activity due to electrostatic interactions (as it increases the number of positively charged residues), while other type of interactions (e.g. hydrophobic) remain unchanged. The proposed competitive ELISA performed in mildy acidic conditions may therefore be useful as a general method for studying LPS interactions, even when future assays must be carried out at physiological pH. Moreover, the assay may be also useful for studying the possible LPS-binding activity of proteins/peptides restricted to certain acidic compartments or environments of cells, tissues and organs [50].

Regarding the differences in the results obtained in competitive hCAS and PMX ELISAs, it should be taken into account that the mechanism of LPS binding to hCAS and PMX is probably different. Thus, in the hCAS-ELISA the interaction with immobilized CAS peptides is predominantly electrostatic (as it increases notably when pH decreases), while for PMX the interaction with LPS seems to be much more complex and of higher affinity. At least two types of bonding are thought to occur in the interaction between PMX with LPS: i) electrostatic bonding between the amino groups of PMX and the phosphate and carboxyl groups of the lipid A-inner core region, and ii) hydrophobic bonding between the hydrophobic side chains of PMX and the acyl lipid chains of LPS [21,49]. It is therefore expected that only molecules with similar affinity and mechanism of interaction with LPS as PMX could prevent PMX-LPS binding while other less selective molecules would be able to inhibit hCAS-LPS binding. In addition, the presence of BSA in the incubation buffer of the competitive PMX-ELISA may also affect the interactions between proteins/peptides and LPS, either by competition or because it disaggregates LPS [51]. The inclusion of BSA in this assay is useful because it produces a stronger signal and less variable results than in its absence (data not shown) and also because, given its abundance in plasma and ability to interact with LPS, it seems a probable candidate to aid in the systemic dissemination of LPS [8].

Except for MYO and P3L, which were tested for the first time, the other molecules investigated in the present study were previously reported to have LPS-binding properties, although the interactions may neutralize or enhance the biological activity of LPS. PMX [48], MF6p [45], LF [41,42] and MEL [46] were reported to be able to bind to and neutralize LPS, which is consistent with the inhibition observed in the competitive ELISAs of the present study. However, HB, which promotes LPS toxicity [11], produced an increase in OD signals in the competitive ELISAs tested. Although there is still some controversy as to whether the active forms of LPS are monomers or multimers with a particular conformation [52], most of the available data attribute the enhanced LPS biological activity to the ability of certain proteins to disaggregate LPS (decrease in the size of aggregates) and/or to promote transition from multilamellar structures to cubic structures [16,25,53]. Our results are consistent with this type of interaction, as the enhanced OD values obtained in the competitive ELISAs are probably due to disaggregation of LPS. However, the results partly contradict some reported data on the LPS-binding properties of two proteins, HF1 and LSZ.

Histones have been proposed as a new class of LPS-binding molecules that are capable of decreasing TNFα production by macrophages [40]. Although the HF1 inhibited binding of both LPS serotypes tested in hCAS-ELISA, the results obtained in PMX-ELISA were inconclusive. Augusto *et al*. [40] tested several histones against LPS from the *Salmonella* Minnesota (rough mutant Re595) but not against smooth strains such as *E*. *coli* O111:B4 and O55:B5 [54]. Thus, it is possible that the interaction is not a general phenomenon but depends on the external O-antigen of some bacteria. Moreover, although the interaction with LPS was demonstrated for histones H1, H2A, H2B, H3 and H4, the capacity to inhibit LPS effects was only tested with H2A.

Regarding LSZ, it has been reported that this protein binds to LPS and can partly suppress LPS-induced TNFα production *in vivo* [43] and *in vitro* [44] under some experimental conditions. In the present study, this protein consistently enhanced the OD values in the hCAS ELISA assay but scarcely caused this effect in the PMX assay and only with the O55:B5 LPS serotype. This suggests that the mechanism of interaction is different from that involving PMX. These results are consistent with some reported data indicating that LSZ may act differently depending on the nature of LPS and does not compete with PMX for LPS binding [29].

In summary, we investigated how several peptides and proteins interacted with LPS under different ELISA conditions and evaluated two competitive methods that may be useful for preliminary screening of large numbers of putative LPS binding molecules. Specifically, we proposed performing the hCAS-ELISA under mildly acidic conditions as a general method for studying LPS interactions, and the more restrictive PMX-ELISA as preferable method for selecting proteins or peptides that are likely to have neutralizing properties *in vitro* or *in vivo*.
